# Precision Therapy for Mesothelioma: Feasibility and New Opportunities

**DOI:** 10.3390/cancers13102347

**Published:** 2021-05-13

**Authors:** Sean Dulloo, Aleksandra Bzura, Dean Anthony Fennell

**Affiliations:** 1 Medical Oncology, University Hospitals of Leicester NHS Trust, Leicester LE1 5WW, UK; sean.dullo@uhl-tr.nhs.uk; 2 Mesothelioma Research Programme, Leicester Cancer Research Centre, University of Leicester, Leicester LE2 7LX, UK; ab973@leicester.ac.uk

**Keywords:** mesothelioma, histotype, Hippo pathway, NF2, BAP1, CDKN2A, PTCH1, SETD2, MTAP

## Abstract

**Simple Summary:**

Mesothelioma remains a lethal cancer. Personalized treatment is lacking. Emerging insights into the genomic and epigenomic landscape of mesothelioma highlight promising opportunities for precision therapy, where are discussed.

**Abstract:**

Malignant pleural mesotheliomas (MPMs) are characterised by their wide variation in natural history, ranging from minimally to highly aggressive, associated with both interpatient and intra-tumour genomic heterogeneity. Recent insights into the nature of this genetic variation, the identification of drivers, and the emergence of novel strategies capable of targeting vulnerabilities that result from the inactivation of key tumour suppressors suggest that new approaches to molecularly strategy therapy for mesothelioma may be feasible.

## 1. Introduction

**Figure 1:**
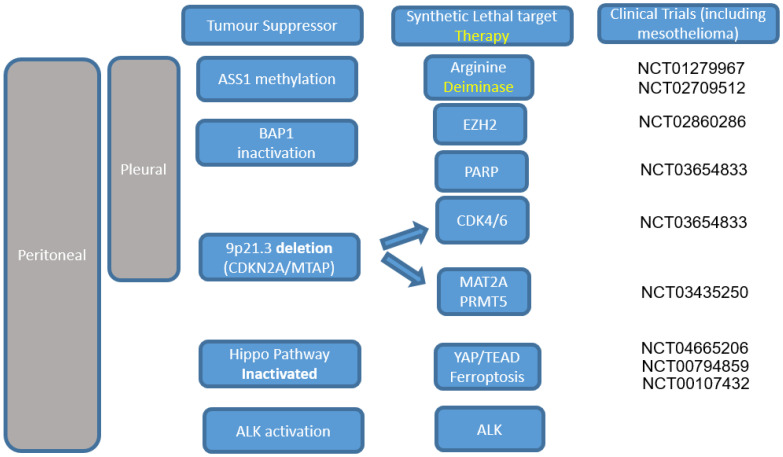
Potentially actionable somatic alterations involving common tumour suppressors in pleural or periotoneal mesothelioma, or oncogene (ALK) in peritoneal mesothelioma. Trials shown on the right are evauating these strategies and are denoted by their trials.gov identifier.

Over the last decade, multiple landmark next-generation sequencing studies of MPM have shed light on the spectrum of recurrently mutated cancer genes [1,2,3,4]. These studies have revealed a preponderance of tumour suppressor gene alterations and dominance of copy number alterations with a relatively low mutation burden of around two mutations per megabase. The absence of a bone fide tyrosine kinase proto-oncogene activating mutations as seen in other cancers (e.g., epidermal growth factor receptor or anaplastic lymphoma kinase, or ROS1 in lung adenocarcinoma), limits the opportunities to target gain-of-function somatic alterations directly. However, emerging insights into the biology of MPM highlight opportunities for targeting vulnerabilities that may emerge due to tumour suppressor inactivation, and potentially, oncogenic processes (Figure 1).

## 2. Histology, Prognosis, and Molecular Stratification of Therapy

To date, the most commonly used classification of MPM has been histopathological, encompassing prognostically distinct subtypes spanning epithelioid (the most frequent and associated with a better prognosis) to biphasic and sarcomatoid (the latter being the most aggressive). Genomic comparisons of these subtypes do not reveal mutually exclusive somatic alterations, with all harbouring, to some extent, the three most common tumour suppressors at 9p21.3 (CDKN2A), 3p21 (BAP1), or 22q (NF2).

However, phenotypically there is a clear gradient of epithelial–mesenchymal transition or EMT [5,6,7] which may underpin chemotherapy resistance and the most aggressive behaviour of mesenchymal-like sarcomatoid MPMs. Patients with sarcomatoid MPM tend to have the worst outcomes with median survival ranging between 3.5 to 8 months [8], considerably shorter than for epithelioid subtype [9]. To date, although EMT exhibits plasticity, targeting EMT to revert a mesenchymal-to-epithelial phenotype has proven to be challenging [10].

The MPM histological spectrum may offer opportunities for stratified therapy. One approach has been to target epithelioid MPMs by taking advantage of the differential expression of mesothelin, which is commonly lacking in sarcomatoid MPMs. For example, the antibody-dependent conjugate aneteumab ravtansine has demonstrated clinical activity [11,12,13] in a molecularly stratified treatment context. Conversely biphasic and sarcomatoid MPMs harbour epigenetic silencing of argininosuccinate synthetase1 (ASS1) which can be therapeutically exploited [9,14,15,16]. ASS1 catalyses the condensation of citrulline with aspartate to form argininosuccinate. Cells that lacking ASS1 expression exhibit a vulnerability to arginine deprivation owing to a dependency (they are unable to convert endogenous citrulline–known as *auxotrophy*). In vitro, deprivation induces apoptosis which translates to clinical efficacy [9,16]. In the clinical trial called Arginine Deaminase and Mesothelioma (ADAM) study, patients were randomised in a 2:1 ratio to ADI-PEG20 (weekly intramuscular dose) versus best supportive care. The primary endpoint was progression-free survival. The trial met its primary endpoint with a superior outcome with a Hazard ratio of 0.56 [9] confirming proof of concept.

A correlation between ASS1, and platinum/antifolate sensitivity was investigated in preclinical models [17,18]. The Phase 1 dose-escalation study involving ADI-PEG 20 in combination with pemetrexed and cisplatin has shown seven (out of nine) MPM patients having partial responses (78%) of which three had sarcomatoid/biphasic histology [14]. This phase 1 subsequently led to the development of the randomised phase 2/3 trial called ATOMIC MESO, randomising ADI-PEG20 or placebo with cisplatin and pemetrexed (ADICiSPem) non-epithelioid MPM patients which most commonly lack ASS1 [19].

## 3. Targeting Hippo Pathway Mutations—Disrupting an Oncogenic Pathway?

One of the most frequent pathways to be inactivated in MPM involves Hippo signalling, a pathway that regulates organ size. The most common somatic alterations involve the neurofibromatosis 2 gene (NF2 22q12) and the large tumour suppressor gene 2 (LATS2, 13q11-12) [20]. NF2 encodes merlin which recruits LATS1/2 kinases which phosphorylate the downstream effectors of the Hippo pathway, yes-associated protein (YAP), and its paralogue TAZ (WW domain-containing transcription regulator 1, or WWTR1). Inhibition of YAP/TAZ prevents their nuclear entry and ability to activate an oncogenic transcriptional programme in partnership with TEA domain transcription factor (TEAD) [21,22]. Therefore, Hippo pathway mutations de-repress a bone fide oncogenic pathway in MPM that is associated with shorter survival.

Recent analysis exploring the evolution of MPM has revealed that Hippo pathway inactivation involving NF2 almost always occurs as a secondary event during early clonal evolution, preceded by another other driver alteration [23]. Using a deep learning methodology to explore phylogenetic data obtained from multiregional sequencing of MPMs, it was repeated evolution was revealed across the cohort. This suggests that Hippo inactivation is deterministic, highlighting its significance as a potential biomarker for novel therapeutic strategies.

Early preclinical studies demonstrated a correlation between merlin loss and upregulation of focal adhesion kinase (FAK); inhibition of FAK was associated with the selective killing of merlin deficient cell lines, highlighting a potential synthetic lethal relationship [24,25]. This concept was then tested in a merlin-stratified, global randomised phase 2 trial called COMMAND [26,27,28], comparing maintenance defactinib or placebo. This study was, however, negative. Further preclinical studies revealed a novel function of FAK as an enhancer of regulatory T cell immunosuppression, leading to a phase 1 trial of defactinib and the PD1 inhibitor pembrolizumab, which includes an MPM cohort [29,30].

Alternative approaches to target Hippo-inactivated MPMs are emerging. Preclinical studies have highlighted potential sensitivity to SRC or BCR/Abl inhibition [31]. TEAD inhibitors are currently in development and could directly disable transcriptional oncogenic signalling [32]. Preclinical studies have identified that Hippo inactivation leads to a vulnerability to ferroptosis, a form of iron-dependent cell death. TEAD signalling upregulates ferroptosis modulators ACSL4 and TFRC, leading to enhanced sensitivity to agents such as sorafenib or sulphasalazine that can modify glutamate transport and cellular redox state [33,34].

## 4. BAP1 Inactivation

BRCA1 associated protein 1(BAP1) is a frequently inactivated tumour suppressor gene in MPM, which is also rarely associated with germline mutation [35]. Mechanisms through which BAP1 inactivation occurs include mutation, copy number loss, or translocations [4]. BAP1 deubiquitinates histone 2A lysine 119, and BAP1 deletion causing an increase in H3K27me3 associated with repression of enhancer of zeste 2 polycomb repressive complex 2 subunit (EZH2) activation. This suggests that small-molecule EZH2 inhibition could be an effective therapy in *BAP1*-mutant cancers. Based on this model, EZH2 inhibition was tested in BAP1 inactivated MPMs [36].

The EZH2 inhibitor tazemetostat was administered to patients with BAP1 inactivated MPM (loss of nuclear expression). The primary endpoint was disease control (i.e., stable disease, complete or partial response) at 12 weeks. The study enrolled 74 patients with the primary endpoint being met at 51%, demonstrating disease control at 12 weeks, with 25% continuing to 24 weeks. Interestingly, 2 of the 61 patients had confirmed partial responses [37]. Based on these data, EZH2 inhibitors might have antitumour activity; however, larger trials would be needed to support these findings.

Nuclear BAP1 regulates homologous recombination (HR) repair via interaction with RAD51 and BRCA1/BARD1 complex [38,39,40], in contrast to its cytoplasmic function in which it modulates calcium signalling mediated cell death [38]. Recruitment to DNA double-strand break sites is mediated via phosphorylation of BAP1, and the role of BAP in DNA damage response involves its catalytic activity [41].

Synthetic lethality associated with BRCA1/2 and PARP inhibition is well established and widely used for targeting homologous recombination deficient cancers. Cells harbouring HR deficiency switch to base excision repair, which is assisted by PARP, to repair DNA single-strand breaks [42]. PARP inhibitors trap PARP on DNA, resulting in catastrophic accumulation of double-strand breaks due to stalling and collapse of DNA replication forks, triggering cell death. The synthetic lethality interaction is observed in the clinic, in tumours harbouring somatic biallelic inactivation in BRCA1/2, leading to approval of PARP inhibitors in BRCA1/2 cancers [43]. A recent panel sequencing study of MPM reported a 36.9% involvement of HR pathway mutations, and this was deemed the most commonly mutated pathway in MPM [44], warranting evaluation of PARP inhibition in MPM.

Recently a phase 2a trial evaluated the use of rucaparib in patients with BAP1 or BRCA1 deficient MPM-*MPM* *Stratified Therapy 1* (MiST1) [45]. The primary endpoint for this trial was 12-week disease control which was met with a disease control rate of 58% (95% CI 37–77) with evidence of durable partial responses lasting more than a year, with manageable toxicity. In another study, olaparib (NCT03531840) reported 81% disease control at 6 weeks, with evidence of partial responses (4%) of which one responder harboured an MRE11A mutation [46]. Niraparib is being explored in patients with Trial (NCT03207347) in BAP1 and other DNA damage repair-deficient neoplasms, including MPM.

Evidence to support BAP1 as a bone fide predictor of sensitivity to PARP inhibition is lacking. One study recently identified that the sensitivity of MPM cells is not dependent on BAP1 but is enhanced by temozolomide in cells with high Schlafen 11 and low O6–methylguanine –DNA methyltransferase expression [47]. On the other hand, a novel MPM-specific splice isoform of BAP1 has been identified, lacking a portion of the catalytic domain, and which had decreased deubiquitinating activity compared to its full-length counterpart [48]. Cells expressing more than 20% of BAP1Δ were found to be more sensitive to olaparib than wild-type BAP1 MPM [48]. Coiled-coil domain containing 6 (CCDC6) interacts with BAP1 and has been reported to regulate both homologous recombination and PARP inhibitor sensitivity. Loss of expression of CCDC6 led to increased preclinical sensitivity to PARP inhibitors and is observed in around 30% of MPMs [32].

PARP inhibitors are proinflammatory and activate cytosolic DNA sensing by cyclic GMP–AMP synthase (cGAS) mediated activation of the endoplasmic reticulum-associated stimulator of interferon genes (STING) pathway [49,50]. The cyclic GMP–AMP synthase/stimulator of IFN genes (cGAS/STING) pathway [51] is responsible for sensing of damaged cytosolic DNA leading to activation of innate immune responses via initiation of signalling cascade involving the cytoplasmic DNA sensor cGAS, in concert with STING and TBK1, and transcription factors, such as IRF3 and NF-κB, that collectively induce a type I IFN response [51]. Therefore, the disruption of nuclear DNA integrity, via endogenous or exogenous factors, activates cGAS/STING pathway, leading to immunotherapy response [52]. Combining PARP inhibitors with immune checkpoint inhibitors in MPM is therefore rational and is being explored in the MIST 5 trial.

## 5. 9p21.3 Deletion

Homozygous deletion of 9p21, the locus harbouring the *p16ink4a* tumour suppressor is a frequent somatic alteration [53]. This deletion occurs within a cluster of genes that include CDKN2B, CDKN2A, and MTAP in up to 72% of MPMs [54]. CDKN2A regulates two important cell cycle proteins p16ink4a (an inhibitor of cyclin-dependent kinases 4 and 6), and p14ARF, an inhibitor of MDM2 which prevents p53 degradation. Restoring p16ink4a function is feasible with small-molecule CDK4/6 inhibition (to phenocopy p16ink4a). Preclinical studies of CDK4/6 inhibitors have reported evidence of nanomolar potency of palbociclib against MPM xenografts [55]. The MIST2 trial has completed accrual testing abemaciclib in p16ink4a negative MPM (results to be presented at the American Society of Clinical Oncology Conference in 2021).

Adenoviral mediated p14ARF gene transfection has been reported to induce G1 cell cycle arrest and apoptosis which was dependent upon the expression of p53 [56]. The heterodimerisation of MDM2 with its homologue, MDMX protein, enhances p53 ubiquitination and degradation. Phase 1 clinical study has investigated AMG 232, a selective MDM2 inhibitor that restores p53 tumour suppression by blocking the MDM2–p53 interaction with picomolar affinity [57] appears to be safe and could provide a strategy to target CDKN2A deleted MPMs, which harbour wild-type p53.

Methylthioadenosine phosphorylase (MTAP), encoded at the 9p21.3 locus is an enzyme essential in the methionine salvage pathway. MTAP converts methylthioadenosine, a product of polyamine synthesis, to adenine and methylthioribose-1-phosphate. The former is used for AMP and the latter for methionine synthesis [54]. MTAP deficiency leads to a dependency on de novo purine synthesis. The first attempt to target MTAP MPM involved L-alanosine (an inhibitor of de novo purine synthesis); however, there were no reported objective responses [58]. However, recently, it has been shown that loss of MTAP leads to elevation of its substrate methylthioadenosine (MTA). This partially inhibits protein arginine methyltransferase 5 (PRMT5) creating a vulnerability to further inhibition [59]. The old antibiotic quinacrine has been recently reported to silence PRMT5 transcriptionally, phenocopying siRNA-mediated inhibition of cell growth [60]. Inhibition of PRMT5 causes defective mRNA splicing and inactivation of MDM4, leading to p53 activation as a major pathway leading to impaired cell growth [61,62]. Interestingly, this pathway is also used by CDK 4/6 inhibitors [63]. An alternative approach being currently explored is the inhibition of MAT2A, the enzyme involved in the synthesis of the PRMT5 substrate, S-adenosyl methionine. MAT2A inhibition appears to be MTAP dependent and also disrupts mRNA splicing. This is approach is being explored in a phase 1 clinical trial with the agent AG-270.

## 6. Anaplastic Lymphoma Kinase (ALK)

ALK rearrangement in NSCLC is well studied and has multiple targeted treatment options with a good prognosis in these patients. In the recent few years, there has been evidence of ALK rearrangements in mesothelioma in the peritoneal subtype. One study that was carried out in pleural MPM identified 25 out of 128 patients (19.5%) with overexpressed ALK transcripts; however, only 10 expressed the ALK protein, and all were negative for ALK rearrangement by fluorescence in situ hybridisation (FISH) [64]. In contrast to the MPM findings, ALK rearrangement tends to be more prevalent in patients with peritoneal MPM, which was confirmed by FISH. ALK positivity was divided into focal weak (no ALK rearrangement) and diffuse strong (ALK rearrangement detected). Sequencing of these samples identified ALK fusion partners STRN, TPM1, ATG16L1 [65]. This has been translated into clinical practice, where the use of ceritinib in a patient with STRN–ALK-rearranged malignant peritoneal mesothelioma showed response as early as 6 weeks into treatment [65].

## 7. PTCH-1

The hedgehog signalling pathway is involved in embryonic development and is inactivated in the adult mesothelium. Hedgehog ligands (Hh) bind to the transmembrane receptor Patched (PTCH1), which subsequently removes the inhibitory influence of the G-protein-coupled receptor smoothened (SMO). SMO activation leads to the induction of glioma-associated protein (GLI 1) and hedgehog interacting protein (Hhip) [66]. PTCH1 has been shown to be positively selected in the MPM [67] suggesting its role as a relatively rare driver (6%). Targeting the Ptch1 could play an important role in targeting the hedgehog signalling pathway. Vismodegib has recently shown activity in a patient with relapsed malignant MPM harbouring PTCH1F1147fs mutation. This patient had a durable response to Vismodegib [68].

## 8. Conclusions

Given the long latency of pleural malignant MPM and ongoing use of asbestos in several non-Western countries, malignant pleural MPM will remain a global health issue during the 21st century. Consequently, there is a pressing need for novel, effective, targeted treatments to improve patient outcomes. Targeted therapy is currently in its infancy for mesothelioma, but emerging developments preclinically and clinically are showing some promise. In summary, targeting altered tumour suppressors in MPM remains a challenge due to the need to identify and action vulnerabilities capable of inducing synthetic lethality; however, promising developments suggest that this may be feasible for the more common somatic alterations in this cancer.

## Data Availability

Not applicable.
